# News and Events of the Week

**Published:** 1910-01-22

**Authors:** 


### NEWS AND EVENTS OF THE WEEK.

MR. J. B. BINNS, Second Resident Assistant Medical Officer at the Infirmary, Bethnal Green, has resigned his post.

ONE of the objects of charity proposed to the trustees of Sir Alfred Jones's will is "original research of all kinds into the cause of disease on the West Coast of Africa.

THE fourth annual report of the National League for Physical Education and Improvement states that the total membership is now 400. Among the new work initiated is the compilation of a London directory of health-promoting agencies and an investigation into the medical inspection of children in institutions such as orphanages, on which a report will be presented. The work of the joint Committee on Physical Education has included a report on physical education in foreign countries, a reprint of which is now ready.

MR. H. T. BUTLIN presided at a quarterly meeting of the Council of the Royal College of Surgeons on the 13th inst., at which the congratulations of the Council were given to Dr. Edgar Jones, of Great Burstead, Brentwood, on his hundredth birthday. Dr. Jones was admitted to the College in 1834. At the same meeting Mr. H. F. Waterhouse was admitted a member of the Court of Examiners, Mr. Edmund Owen was appointed Hunterian Orator for 1911, and Mr. Pearce Gould was appointed Bradshaw Lecturer for the ensuing collegiate year.

THE Central Poor Law Conference will be held at the Guildhall on Tuesday, February 22, and following day. The Lord Mayor will open the proceedings, over which Lord Richard Cavendish will preside. The principal subject for discussion will be the Minority Report of the Royal Commission on the Poor Laws and Relief of Distress. Mr. Vulliamy (Ipswich) will open the question, and papers on Poor Law relief work will be read by Mr. James Blossom (Hon. Secretary of the North Midland District Conference) and Dr. Parsons (Fulham). Delegates will be sent by boards of guardians in all parts of England and Wales, and the Metropolitan Poor Law Guardians will also be represented.

ACCORDING to returns from the Metropolitan Asylums Board and from the London Fever Hospital there were five cases of smallpox, 2,256 of scarlet fever, 908 of diphtheria, and 56 of enteric fever under treatment in these hospitals on Saturday, January 8. The admissions during the week, as compared with those in the preceding three weeks, were as follows: Of smallpox, two cases, against one, one, and one; of scarlet fever, 212 cases, against 254, 224, and 244; of diphtheria, 108 cases, against 99, 88, and 85; and of enteric fever, 11 cases, against eight, eight, and nine.

THE latest weekly return of births and deaths in London and in the 75 other great towns of England and Wales, issued by authority of the Registrar-General, states that the deaths registered last week corresponded to an annual rate of 14.8 per 1,000 of their aggregate population, which is estimated at 16,713,617 persons in the middle of last year. In the preceding three weeks the rates had been 15.0, 15.4, and 16.7. Measured by last week's mortality, the highest annual death rates per 1,000 living were: From all causes, 20.4 in Nottingham, 21.1 in Rhondda, 21.3 in West Bromwich, 22.3 in Stockton-on-Tees, 22.9 in Huddersfield, 23.4 in Birkenhead, 23.5 in Warrington, and 25.4 in Hanley; from measles, 1.6 in Swansea and 1.7 in Birkenhead; and from whooping-cough, 1.7 in Plymouth.

THE report of the medical officer of the Local Government Board for 1908-9 has just been issued. Dr. Arthur Newsholme, who has prepared it, remarks that the general death-rate for 1908 was the low one of 14.7, but that our satisfaction is much spoiled because 14.7 represents not a standard but an average.

MR. K. W. MONSARRAT, M.B., C.M., F.R.C.S., lecturer in clinical surgery in the University of Liverpool, will deliver a public lecture on "The Conditions Which Determine Predominance Among Individuals and Species," in the Arts Theatre of the University at 8 P.M., on February 11.

WE have received the second edition of Messrs. J. and A. Churchill's excellent illustrated catalogue of medical books. The first edition was so favourably received that it was reprinted twice in three weeks. The second edition is an improved and extended catalogue, which should prove very useful to medical men.

IN a long letter to the *Times* of the 18th inst. on quinine prophylaxis, "Surgeon Lieut.-Colonel, I.M.S." urges on the medical profession the importance of agreeing how and to what extent quinine should be administered in malaria and blackwater fever. He adds that, although Manson's *Tropical Diseases* says that quinine will cure 99 per cent. of such cases, medical disagreement has so infected the public mind that nobody he could meet with on the Gold Coast, who used quinine as a prophylactic, took more than five grains a day, and many refused to regard it even as a palliative. Till the distant day when the country's revenue admits of sanitary measures on a proper scale, quinine prophylaxis, he says, is the only method on which any reliance can be placed.

THE Manchester Industrial Exhibition will be held May 12 to June 25 next, 1910. It will be the first important industrial exhibition that has been held in Manchester for over twenty-two years. The exhibition buildings, which are now in course of erection, possess double the available ground floor area of the largest building in any provincial city, the ground floor space being in excess of that of the Royal Agricultural Hall, London, and having an area of approximately 100,000 super. feet, exclusive of the subsidiary halls. The exhibition will be of a comprehensive and high-class character. Special sections will be devoted to exhibits of the building and kindred trades and of everything conducive to the comfort and beauty of home life.

AT a meeting representing the Royal Colleges of Physicians and Surgeons in Ireland with members of Parliament and the Unionist candidates for the City of Dublin on January 15, Mr. John Lentaigne, President of the College of Surgeons, said that the school of that college was at present the only school of the kind in Ireland entirely unsupported by Parliament. Other schools now competing with it were State endowed. If relief were not quickly given it must die, and if their school died so also would the Royal Colleges of Surgeons and Physicians. Dr. Horne, President of the College of Physicians, said that owing to the establishment of the new University they would not be able in future to compete with the University school unless the Government came to their assistance. Sir Edward Carson, M.P., said that he and his colleagues would join with their Nationalist opponents in pressing upon the Government the claims of the colleges.

The will of the late Dr. John Waggett, M.D.(Edin.), of Perrivale, Bournemouth, who died recently, in his 91st year, has been proved at £50,184 gross and £49,542 net.

Dr. Bartlet was elected last week President of the East Suffolk and Ipswich Hospital by the Court of Governors in succession to Mr. Felix Cobbold.

During the week ending January 5 ten cases of deaths by poisoning were recorded, four being suicidal. In two suicidal cases ammonia was the poison selected, in one laudanum, and in one strychnine.

Dr. Henry Robinson made a startling statement at a recent meeting of the Hull Corporation Sanitary Committee. He said that to his knowledge, a few weeks ago, there had been a carcass infected with anthrax in a Hull butcher's shop, and the whole of it was consumed by the public. The Deputy-Chairman of the Committee (Mr. Raine), commenting upon the gravity of the statement, said that Dr. Robinson failed in his duty in not before drawing the attention of the Committee to the case. Dr. Robinson said he did not know it at the time; he was told afterwards, and he could prove his words.

The College of Physicians of Philadelphia announce that the next award of the Alvarenga prize, being the income for one year of the bequest of the late Señor Alvarenga, and amounting to about 180 dollars (£36), will be made on July 14, 1910, provided that an essay deemed by the committee of award to be worthy of the prize shall have been offered. Essays for the competition may be upon any subject in medicine, but must not previously have been published. They must be typewritten, and must be received by the Secretary of the College on or before May 1, 1910. Each essay must be sent without signature, but must be plainly marked with a motto and be accompanied by a sealed envelope having on its outside the motto and within the name and address of the author. The successful essay, or a copy of it, will remain in possession of the College; other essays will be returned upon application within three months after the award.

